# Negative-Viscosity Materials: Exploiting the Effect of Negative Mass

**DOI:** 10.3390/ma18061199

**Published:** 2025-03-07

**Authors:** Edward Bormashenko, Shraga Shoval

**Affiliations:** 1 Department of Chemical Engineering, Bio-Technology and Materials, Engineering Faculty, Ariel University, Ariel 407000, Israel; 2 Department of Industrial Engineering and Management, Faculty of Engineering, Ariel University, P.O. Box 3, Ariel 407000, Israel; shraga@ariel.ac.il

**Keywords:** metamaterials, negative viscosity, core–shell systems, frequency, Newtonian liquid, drag force, plasma oscillations

## Abstract

The research is motivated by the search for materials with negative viscosity to exploit the effect of negative mass. We introduce media (gaseous and liquid) that demonstrate negative viscosity. Consider the vibrated plate, which is vertically pulled through the ideal gas and built from the core–shell “meta-molecules”. Vibrating the vertical plate supplies an excess vertical momentum to the core–shell meta-molecules. If the frequency of vibrations is larger than the resonant frequency, the excess moment is oriented against the direction of the vertical motion; thus, the effect of negative viscosity becomes possible. The effective viscosity becomes negative when the frequency of the plate vibrations approaches the resonant frequency from above. Thus, a novel physical mechanism resulting in negative viscosity is introduced. No violation of energy conservation is observed; the energy is supplied to the system by the external source vibrating the plate. The effect of the negative viscosity is also possible in liquids. Frequency dependence of the viscosity is addressed. Asymptotic expressions are derived for the frequency-dependent viscosity. Introduced meta-materials may be exploited for the development of media with prescribed rheological properties. Possible realizations of the negative-viscosity media are discussed.

## 1. Introduction

The development of optical and acoustic metamaterials opened new horizons in materials science [1,2,3]. The history of metamaterials started from the seminal paper by Victor Veselago, who, in 1968, first theorized that there were materials with negative refractive indices, a fundamental property of optical metamaterials, in his seminal paper on left-handed materials [1]. The first experimental realizations of optical metamaterials were reported only thirty years later [2]. Metamaterials are usually defined as artificial, periodic structures suited for control/altering mechanics [2] or electromagnetic [1,4] properties of materials to obtain prescribed features that are not observed naturally. The prefix “μετά” (a Greek word meaning ‘beyond, after’) indicates that the properties of the material are outside of the range of properties observed in nature [2,3,4,5,6,7,8,9,10,11,12,13,14]. The structural units of the metamaterials (“meta-molecules”) are usually macroscopic [2,3,4,5,6,7,8,9,10,11,12,13,14]. These macroscopic meta-molecules can be tailored and dynamically controlled in shape, properties, chirality and size [2,3,4,5,6,7,8,9,10,11,12,13]. The lattice constant and inter-cell interaction can be artificially modified, and useful “defects” can be designed and introduced precisely at desired locations [8,9,10]. Metamaterials supplied to engineers media with the properties, which do not exist in the natural materials and alloys.

The electrical permittivity and the magnetic permeability are the main determinants of a material’s response to electromagnetic waves. In metamaterials, both these material parameters are negative [7,8,9,10]. It was Victor Veselago who noted the solutions of the Maxwell equations, assuming negative electrical and magnetic permittivity, are possible [1]. Another fascinating property of metamaterials is the reverse Doppler effect [12]. Left-handed metamaterials have already been elaborated and enabled numerous applications, including invisible cloaks, EM concentrators, beam splitters and new types of antennas [2,5].

Numerous amazing, paradoxical and useful effects, such as the negative effective mass, became possible with the meta-materials [15,16,17,18,19,20,21,22,23,24,25]. The effect of negative effective mass means that applying a force on a resonant mechanical system would result in certain conditions of acceleration in the opposite direction of the force [15]. The effect of the negative effective mass (also called “negative inertia” [25]) may be achieved by exploiting the plasma oscillations of the electron gas in metals [21,22]. The negative effective mass materials were already successfully demonstrated experimentally by embedding soft silicon rubber-coated heavy spheres in a thermosetting polymer, acting as the local mechanical resonators [24]. The development of metamaterials enabled unique engineering applications, including seismic devices, optical and acoustic camouflage and acoustic and thermal cloaking/absorbers [8,9,10,26,27,28,29]. Metamaterials enable the achievement of controlled and exotic mechanical properties through the rational design of their microstructures. It was demonstrated recently that when compared with conventional schemes, metamaterials provide unprecedented potential for governing the mass and energy transport processes [3,12]. We report in this communication that the paradoxical control of momentum transport, which results in negative viscosity emerging from the effect of the negative mass, is possible. A “negative-viscosity” effect in a magnetic fluid was already reported [30,31]. In a Poiseuille flow, a constant magnetic field balances vorticity and impedes the rotation of individual magnetic particles embedded into the liquid [30,31]. Conversely, an alternating magnetic field favors the rotation of the particle [30,31]. The magnetic energy is transformed into the angular momentum of the magnetic particles, which is converted into a hydrodynamic motion of the fluid [30,31]. The entire effect results in a decrease in the total viscosity [30]. The negative viscosity of ferrofluids under alternating magnetic fields was reported by other groups [32,33]. Negative effective viscosity also emerges in a large-scale flow maintained by a small-scale periodic force field [34]. If the field is essentially anisotropic, the system of small-scale eddies generated in the liquid is unstable to long-wave disturbances, i.e., the effective viscosity of the corresponding large-scale flow is negative [34]. If the applied periodic field is isotropic, the long-wave instability disappears; thus, the effective viscosity is positive, as reported by Sivashinsky and Yakhot [34]. We introduce the additional possible mechanism of negative viscosity, exploiting the effect of negative mass.

## 2. Results

**Figure 1:**
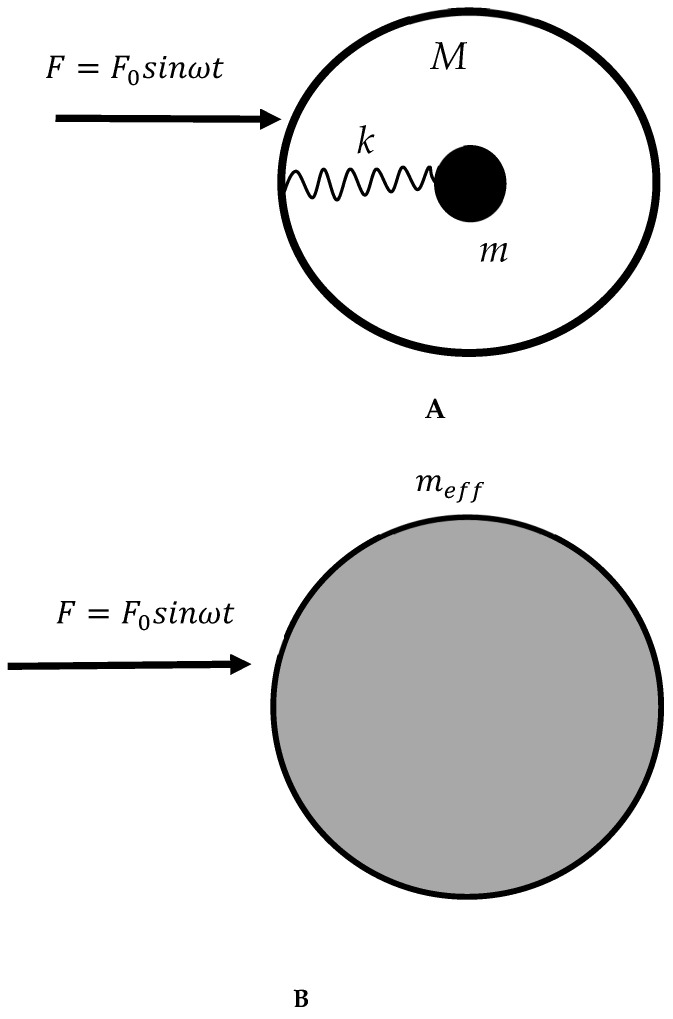
(**A**) The core–shell unit exerted on the sinusoidal external force is presented. The mass of the shell is *M*, and the mass of the core is *m*. The core particle *m* is connected to the spherical shell *M* with the ideal Hookean, massless spring *k*. (**B**) The core–shell unit is replaced by the single body with a mass of $m_{eff}.$

Let us introduce the effect of the negative mass. Consider, first, the core–shell unit depicted in Figure 1A. The mass of the shell is *M*, and the mass of the core is *m*. The core particle *m* is connected to the spherical shell *M* with the ideal Hookean, massless spring *k*. Assume that the harmonic external force acting on the entire system is $F\left(t\right)=F_{0}sin\omega t$, represented by the complex force $\hat{F}\left(t\right)={\hat{F}}_{0}e^{j\omega t}$. It was demonstrated that the core–shell unit might be substituted by a single mass, denoted as $m_{eff}$, which performs the same motion as shell *M,* as depicted in Figure 1B [15,16,17,18]. In other words, the core mass *m* is ignored, and the core–shell system is replaced by the single mass $m_{eff}$, as shown in Figure 1B, which moves with the complex amplitude $\hat{X}$:

(1) $$\hat{X}=\frac{{\hat{F}}_{0}}{m_{eff}\omega^{2}},$$

where an equivalent effective mass, denoted as $m_{eff}$, is supplied with Equation (2) (for details on the derivation of Equation (2), see [15,16,17,18]):

(2) $$m_{eff}=M+\frac{m\omega_{0}^{2}}{\omega_{0}^{2}-\omega^{2}},$$

where $\omega_{0}=\sqrt{\frac{k}{m}}$ is the resonance frequency. It is easily recognizable from Equation (2) that when the frequency of the external harmonic force ω approaches $\omega_{0}$ from above the effective mass $m_{eff}$, as defined by Equation (2), it will be negative. The effect was also called “negative inertia” [25]. Of course, actually, neither “negative mass” nor “negative inertia” exists, and this should be clearly understood. What does the effect of negative mass actually mean? It means that under certain frequencies, the entire core–shell system, which is seen as an integral unit, moves in the opposite direction of the external force (in other words, the core–shell unit moves against the applied harmonic force) [15,16,17,18].

**Figure 2:**
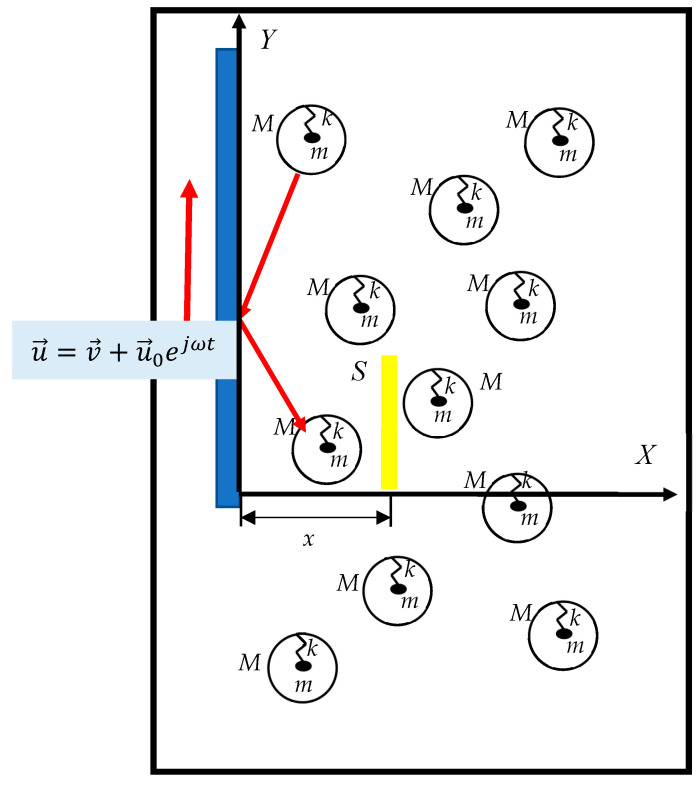
The blue plate is vertically pulled through the ideal gas of the core–shell system with the velocity $\vec{u}={\vec{v}+\vec{u}}_{0}e^{j\omega t};\;\left|\vec{v}=const\right|$<<$\left|{\vec{u}}_{0}=const\right|$. Axis *X* is in a normal orientation with the plate. A transverse transfer of momentum through the cross-section S (shown with a yellow rectangle) is considered.

Consider now the ideal gas built from core–shell particles/units, as shown in Figure 2. We assume that the core–shell units are not subject to inter-particle interactions. Core–shell units participate in the random thermal motion (we will restrict the randomness of this motion below). The core–shell units, considered in our approach as “meta-molecules”, give rise to the effect of the negative mass.

Consider the vertical plate pulled vertically upward (along axis *Y*) through the gas of core–shell particles (i.e., meta-molecules) with the velocity $\vec{u}={\vec{v}+\vec{u}}_{0}e^{j\omega t};\;\left|\vec{v}=const\right|$<<$\left|{\vec{u}}_{0}=const\right|$, as shown in Figure 2. For the sake of simplicity, we assume the vibration of the core masses *m* occurs along axis *Y*; i.e., the core–shell units are oriented vertically (the springs remain oriented vertically). Vibrating vertical plate supplies to the core–shell units an excess vertical momentum. If $\omega <\omega_{0}$, this excess momentum coincides with the positive direction of axis *Y*; however, if $\omega >\omega_{0}$, the excess moment is oriented against axis *Y* $\;(\left|\vec{v}\right|$<<$\left|{\vec{u}}_{0}\right|$ is adopted); thus, the effect of negative viscosity becomes possible. Consider the case when $\omega >\omega_{0}$ is true. The vibrated plate supplies a downward momentum to the core–shell meta-molecules; thus, the gas of core–shell units supplies an excess upward moment to the plate. This effect may be described as the negative viscosity of the gas built from the core–shell elements.

We further will address the ideal gas of the core–shell particles possessing the number concentration of *n*. Particles participate in the random thermal motion with a 3D average velocity *c* (the motion of the particles is not completely random: the vertical orientation of the springs remains untouched). Consider the transfer of excess vertical moment ∆p  supplied by the vertically pulled plate ($\vec{u}={\vec{v}+\vec{u}}_{0}e^{j\omega t}$) to the gas, built from the core–shell particles, through the vertical area *S*, located at the distance *x* from the plate (see Figure 2) over the time interval ∆t. This transfer equals

(3) $$∆p=m_{eff}v_{1}∆N_{+}-m_{eff}v_{2}{∆N}_{-}=\frac{1}{6}m_{eff}nSc∆t\left(v_{1}-v_{2}\right)=\frac{2\lambda }{6}m_{eff}nSc\Delta t\frac{v_{1}-v_{2}}{2\lambda },$$

where $m_{eff}$ is supplied by Equation (2); $v_{1}$ and $v_{2}$ are the velocities of excess, oriented, vertical motion of the core–shell units before and after passing area *S*; $∆N_{+}$ and ${∆N}_{-}$ are the number of core–shell units passing through the area along and against the positive direction of axis *x* correspondingly; and λ  is a mean free path of core–shell units. The excess momentum is transferred to the other molecules via collisions. Newton’s Second Law yields for the viscous stress of Equation (3):

(4) $$\tau_{visc}=\frac{\Delta p}{S\Delta t}=-\frac{1}{3}m_{eff}\lambda nc\frac{\partial v}{\partial x}=-\eta_{eff}\frac{\partial v}{\partial x},$$

where $\eta_{eff}=\frac{1}{3}m_{eff}\lambda nc$ is the effective viscosity of the core–shell units’ built gas. Considering Equation (2) and $c=\sqrt{\frac{8k_{B}T}{\pi \left(m+M\right)}}$ (*T* is the temperature of the core–shell units built gas) yields for the effective viscosity,

(5) $$\eta_{eff}\left(\omega \right)=\frac{1}{3}(M+\frac{m\omega_{0}^{2}}{\omega_{0}^{2}-\omega^{2}})\lambda n\sqrt{\frac{8\pi k_{B}T}{\pi \left(m+M\right)}}$$

The effective viscosity $\eta_{eff}$ given by Equation (5) is negative when the frequency of the external harmonic force vibrating the plate ω approaches resonance frequency $\omega_{0}$ from above. It should be emphasized that the effective viscosity supplied by Equation (5) is frequency-dependent. Such a behavior is typical for non-Newtonian liquids [35,36].

**Figure 3:**
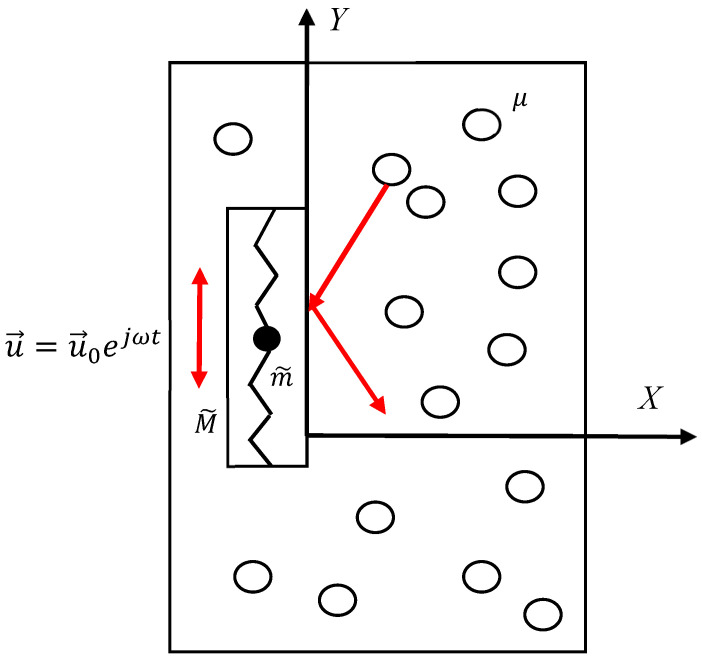
The core–shell system oscillates vertically when embedded into the ideal gas of the particles with mass μ.

Now consider one more possibility of the negative effective viscosity emerging in the situation illustrated in Figure 3. Consider the core–shell system, which is oscillated vertically within the ideal gas with the velocity with the velocity $\vec{u}={\vec{u}}_{0}e^{j\omega t};\;\left|{\vec{u}}_{0}\right|=const$; the mass of the shell is $\tilde{M}$; the mass of the core is $\tilde{m}$; and the core mass $\tilde{m}$ is connected to the shell with two ideal, massless Hookean springs $\frac{k}{2}$. The core–shell is embedded into the ideal gas of the particles with a mass of μ and concentration of *n*. The particles move with an average velocity of $c=\sqrt{\frac{8k_{B}T}{\mu m}}$. Obviously, the transverse transport of momentum occurs with the classical viscosity, given by $\eta =\frac{1}{3}\mu \lambda nc$, where λ  is a mean free path of the particles μ. So, this case looks trivial, and it does not give rise to the phenomenon of negative viscosity. However, the actual situation is more complicated. Consider the drag force $F_{drag};$ we address the situation when the Reynolds number is much smaller than the unity ($Re=\frac{\rho u_{0}a}{\eta }\ll 1,\;where\;\rho$ is the density of the gas, and a is the characteristic dimension of the core–shell system). Thus, the drag force may be expressed as follows [35]:

(6) $$\left|F_{drag}\right|=\varrho a\eta \dot{y},$$

where ϱ is the dimensionless coefficient, and axis *Y* is vertical (see Figure 3). The second Newton Law yields the following:

(7) $$m_{eff}\ddot{y}=-\varrho a\eta \dot{y},$$

where $m_{eff}=\tilde{M}+\frac{\tilde{m}\omega_{0}^{2}}{\omega_{0}^{2}-\omega^{2}}$; $\omega_{0}=\sqrt{\frac{k}{\tilde{m}}}$. Considering $\dot{y}(t)=\left|u_{0}\right|e^{j\omega t}$ yields the following:

(8) $$\left(\tilde{M}+\frac{\tilde{m}\omega_{0}^{2}}{\omega_{0}^{2}-\omega^{2}}\right)j\omega =-\varrho a\eta$$

Equation (7) enables the introduction of effective complex viscosity with Equation (8):

(9) $$\eta_{eff}=\frac{1}{\varrho a}\left(\tilde{M}+\frac{\tilde{m}\omega_{0}^{2}}{\omega_{0}^{2}-\omega^{2}}\right)j\omega$$

Thus, we obtain the following:

(10) $${Im\eta }_{eff}=\frac{1}{\varrho a}\left(\tilde{M}+\frac{\tilde{m}\omega_{0}^{2}}{\omega_{0}^{2}-\omega^{2}}\right)\omega$$

**Figure 4:**
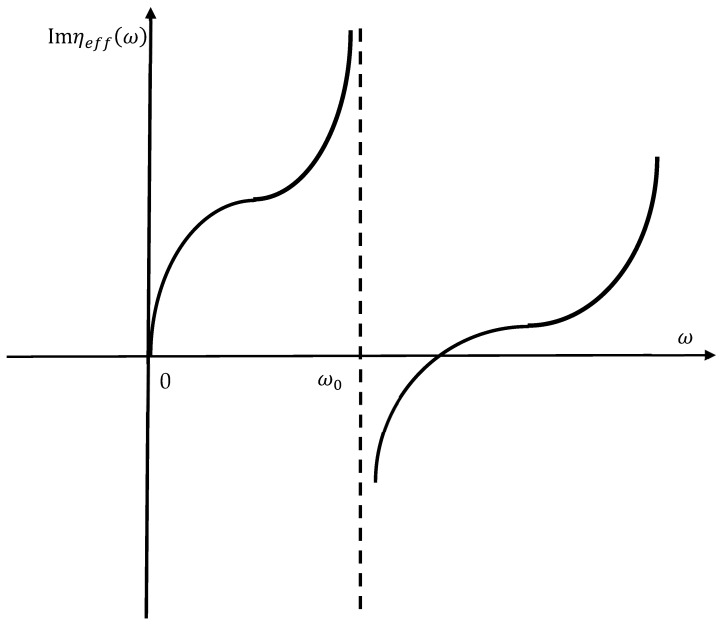
The dependence $Im\eta_{eff}\left(\omega \right)$ established for the system shown in Figure 3 is depicted (see Equation (10)).

It is instructive to build the graph ${Im\eta }_{eff}(\omega )$; such a graph is schematically shown in Figure 4. It is easy to demonstrate that the function ${Im\eta }_{eff}(\omega )$ has no extremal points. It is also easy to see that ${Im\eta }_{eff}\left(\omega \right)<0$ takes place when ω approaches the resonant frequency $\omega_{0}$ from above (see Figure 4).

The same approach may be applied to the core–shell system (the mass of the shell is $\tilde{M}$, the mass of the core is $\tilde{m}$, the core is connected to the shell with the ideal spring k) embedded into the Newtonian liquid (a Newtonian liquid is a fluid that follows Newton’s law of viscosity, which states that the shear stress is directly proportional to the shear rate) η,  as depicted in Figure 4. The core–shell system is oscillated horizontally with the velocity $\vec{u}\left(t\right)={\vec{u}}_{0}e^{j\omega t};\;\left|{\vec{u}}_{0}\right|=const$. In this case, Newton’s Second Law yields the following (we assume that the Reynolds number is much smaller than the unity):

(11) $$m_{eff}\ddot{x}=-6\pi \eta R\dot{x},$$

where $F_{drag}=-6\pi \eta R\dot{x}$ is the Stokes drag force, $m_{eff}=\tilde{M}+\frac{\tilde{m}\omega_{0}^{2}}{\omega_{0}^{2}-\omega^{2}}$, and $\omega_{0}=\sqrt{\frac{k}{\tilde{m}}}$. Considering $\dot{x}(t)=\left|u_{0}\right|e^{j\omega t}$ gives rise to the following:

(12) $$\left(\tilde{M}+\frac{\tilde{m}\omega_{0}^{2}}{\omega_{0}^{2}-\omega^{2}}\right)j\omega =-6\pi \eta R$$

Thus, we obtain the following for the effective viscosity:

(13) $$\eta_{eff}=\frac{1}{6\pi R}\left(\tilde{M}+\frac{\tilde{m}\omega_{0}^{2}}{\omega_{0}^{2}-\omega^{2}}\right)j\omega$$

The imaginary part of the effective viscosity is supplied with Equation (14):

(14) $${Im\eta }_{eff}\left(\omega \right)=\frac{1}{6\pi R}\left(\tilde{M}+\frac{\tilde{m}\omega_{0}^{2}}{\omega_{0}^{2}-\omega^{2}}\right)\omega$$

**Figure 5:**
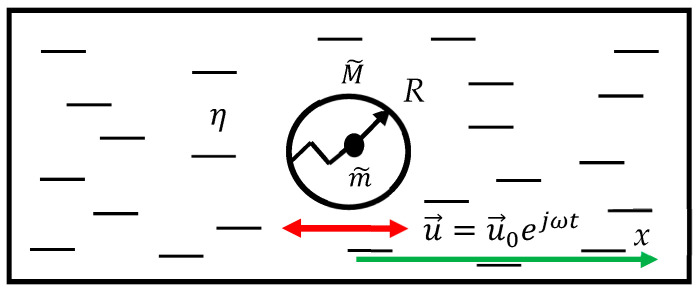
The core–shell system embedded into the liquid with viscosity η. The mass of the shell is $\tilde{M}$, the mass of the core is $\tilde{m}$, and the core is connected to the shell with the ideal spring k. The core–shell system is oscillated horizontally within the liquid η with the velocity with the velocity $\vec{u}={\vec{u}}_{0}e^{j\omega t};\;\left|{\vec{u}}_{0}\right|=const$. The radius of the shell is *R* (shown with arrow).

Plot ${Im\eta }_{eff}\left(\omega \right)$ coincides qualitatively with that shown in Figure 4. It should be emphasized that expressions (9) and (13), which supply the effective viscosity of the media, have nothing to do with the true viscosities of the gas or liquid. Indeed, the viscosity of the media does not depend on the mass of the body, which moves through these media. However, these expressions are useful for understanding the frequency dependence of the drag force, as illustrated in Figure 5. It is instructive to supply the asymptotic expressions for ${Im\eta }_{eff}\left(\omega \right)$. In the limit of high frequencies, we calculate the following:

(15) $$\underset{\omega \to \infty }{\lim}Im\eta_{eff}\left(\omega \right)=\frac{\tilde{M}\omega }{6\pi R}$$

In the limit of low frequencies, we obtain the following in turn:

(16) $$\underset{\omega \to 0}{\lim}Im\eta_{eff}\left(\omega \right)=\frac{(\tilde{M}+\tilde{m})\omega }{6\pi R}$$

It should be emphasized that the frequency dependence of effective viscosity, described by Equations (13)–(16), stems from the resonant properties of the core–shell system and not from the properties of the liquid, which are supposed to be Newtonian.

## 3. Discussion

The goals of this study are defined as follows:

(i) Introducing of gaseous medium, demonstrating negative viscosity and exploiting resonant core–shell systems;
(ii) Introducing liquid media, demonstrating negative effective viscosity and exploiting the resonant negative effective mass of the vibrated body moving through the liquid.

The counterintuitive, paradoxical negative viscosity effect has already been discussed in the literature. This effect occurs in a Poiseuille flow of a ferrofluid when a constant magnetic field balances vorticity and impedes the rotation of individual magnetic particles embedded into the liquid [30,31]. Negative viscosity also emerges in ferrofluids under alternating magnetic fields [32,33]. It is also possible in a large-scale flow maintained by a small-scale periodic force field [34]. We introduce the novel mechanism of negative viscosity, which occurs in the media built from the core–shell systems, introduced in [15,16,18,19]. Consider the vibrated plate, which is pulled through the gas and built from the core–shell systems, which are seen as meta-molecules. The effective viscosity $\eta_{eff}$ is negative when the frequency of the external harmonic force vibrating the plate ω approaches $\omega_{0}$ from above. It should be emphasized that the negative viscosity does not violate the conservation of energy; indeed, the plate is vibrated by the external source of energy.

The effect of negative viscosity may be useful for the following:

(i) Intelligent control of the self-sustained vibrations [37];
(ii) Flutter suppression in turbine engineering. The efficiency of turbines depends strongly on the Reynolds of the gas flow [38,39];
(iii) Development of smart and active materials with controlled rheology properties [40];
(iv) Energy injection in epithelial cell monolayers [41].

Now consider the possible experimental realization of the suggested model gas. The spring connecting the core mass to the shell should not necessarily be the mechanical one (see Figure 1). Actually, the “spring” may represent the plasma oscillations of the electron gas. Plasma oscillations of ionized air and $N_{2}$ gases were registered in the THz band [42,43]. Thus, the effect of negative mass is expected for vibrated bodies moving in gaseous plasmas when the frequency of vibration is higher than the plasma frequency, denoted as $\omega_{p}$. Consider that THz acoustic vibrations are already available [44].

One more realization of a negative-viscosity medium is possible. We have already introduced the negative effective mass meta-material based on the electro-mechanical coupling of the plasma oscillations of a free electron gas immersed in a periodic lattice of fixed positively charged ions [21,22,45]. The negative effective mass appeared as a result of the excitation of a semiconductor particle with an external frequency ω, which is close to the frequency of the plasma oscillations of the electron gas *m*, labeled $\omega_{p},$ relative to the immobile ionic lattice *M*, which is again located in the THz band for some of the semiconductors. The plasma oscillations of the electron gas are described with the elastic spring constant $k=\omega_{p}^{2}m$. Thus, the semiconductor particle excited with the external electric field of frequency ω is substituted by the effective mass $m_{eff}=M+\frac{m\omega_{p}^{2}}{\omega_{p}^{2}-\omega^{2}}$, which becomes negative when the excitation frequency comes to $\omega_{p}$ from above. Thus, the introduced negative-viscosity gas may be implemented with the gas of semiconductor particles exerted on the external harmonic electromagnetic field [46]. Perhaps the nearest candidate for such an implementation is a dusty plasma composed of negatively charged dust grains and ions [47]. Regardless, the plasma oscillations of the effect are the high-frequency (THz) ones, and the low-frequency realizations of the phenomenon should be developed in future research.

## 4. Conclusions

Our paper introduces a novel approach to the problem of negative viscosity based on the resonance in the core–shell systems. The concept of negative viscosity is well-rooted in hydrodynamics and astrophysics. In universe models dominated by negative viscosity, the fluid’s entropy decreases with time, as one would expect [48]. In astrophysical accretion disks (around black holes and stars), negative viscosity can help explain how angular momentum is transferred outward, facilitating accretion [49]. It was suggested that negative viscosity influences the wind-driven, barotropic ocean circulation in a subtropical basin [50]. Negative effective viscosity also appears in a large-scale flow governed by a small-scale periodic force field [34].

We introduce the metamaterials demonstrating the negative viscosity based on the phenomenon of negative effective mass. The effect of negative effective mass emerges in core–shell systems exerted on the harmonic force [15,16,17,18,19]. It was demonstrated that under certain frequencies, the entire core–shell system, which is seen as an integral unit, moves in the opposite direction of the external force [15,16,17,18,19]. The core–shell unit may be substituted by a single “negative” effective mass, which moves against the applied harmonic force, as shown in Figure 1. Negative mass/density materials were already successfully applied for the damping of vibrations in mechanical engineering and the building industry [51,52,53].

We addressed the motion of the vibrated plate through the ideal gas built from non-interacting “quasi-molecules” (i.e., “meta-molecules”), which are represented by the core–shell systems, in which the core mass *m* is connected to the shell *M* with the ideal massless spring *k*. The following model system was suggested: Consider the vibrated plate, which is vertically pulled through the gas and built from the “meta-molecules”/core–shell systems. *Y* is the vertical axis. Vibrating the vertical plate supplies the core–shell “meta-molecules” with excess vertical momentum. If $\omega <\omega_{0}$ ($\omega_{0}=\sqrt{\frac{k}{m}}$), this excess vertical component of the momentum coincides with the positive direction of axis *Y*; if $\omega >\omega_{0}$, the excess moment is oriented against axis *Y.* Thus, the effect of negative viscosity becomes possible. It should be stressed that no violation of energy conservation is observed; the energy is supplied to the system by the external source vibrating the plate. The effect of the negative viscosity is also possible in liquids. We also considered the core–shell system embedded in the Newtonian liquid in which the Stokes drag force acts on the vibrated core–shell unit. The entire system demonstrates the effect of the negative viscosity. The frequency dependence of the viscosity is addressed. Low- and high-frequency asymptotic expressions for the frequency-dependent viscosity are derived. Introduced model meta-materials may be exploited for the development of materials with smart, counterintuitive rheology properties. Realizations of the negative-viscosity materials, exploiting plasma oscillations as a spring, are introduced. Negative viscosity may be exploited for the intelligent control of self-sustained vibrations, including flutter suppression. Negative viscosity, as an effect of active flow control, can be used to reduce drag in pipelines, airfoils and marine vehicles. Negative viscosity effects can enhance turbulence-driven transport in magnetized plasmas, influencing confinement strategies in tokamaks and stellarators. Additional low-frequency realizations of the effect should be developed in future research.

## Data Availability

The original contributions presented in this study are included in the article. Further inquiries can be directed to the corresponding author.
